# Improved region of interest selection and colocalization analysis in three-dimensional fluorescence microscopy samples using virtual reality

**DOI:** 10.1371/journal.pone.0201965

**Published:** 2018-08-29

**Authors:** Rensu P. Theart, Ben Loos, Yigael S. L. Powrie, Thomas R. Niesler

**Affiliations:** 1 Department of Electrical and Electronic Engineering, Stellenbosch University, Stellenbosch, Western Cape, South Africa; 2 Department of Physiological Sciences, Stellenbosch University, Stellenbosch, Western Cape, South Africa; Istituto Italiano di Tecnologia, ITALY

## Abstract

Although modern fluorescence microscopy produces detailed three-dimensional (3D) datasets, colocalization analysis and region of interest (ROI) selection is most commonly performed two-dimensionally (2D) using maximum intensity projections (MIP). However, these 2D projections exclude much of the available data. Furthermore, 2D ROI selections cannot adequately select complex 3D structures which may inadvertently lead to either the exclusion of relevant or the inclusion of irrelevant data points, consequently affecting the accuracy of the colocalization analysis. Using a virtual reality (VR) enabled system, we demonstrate that 3D visualization, sample interrogation and analysis can be achieved in a highly controlled and precise manner. We calculate several key colocalization metrics using both 2D and 3D derived super-resolved structured illumination-based data sets. Using a neuronal injury model, we investigate the change in colocalization between Tau and acetylated *α*-tubulin at control conditions, after 6 hours and again after 24 hours. We demonstrate that performing colocalization analysis in 3D enhances its sensitivity, leading to a greater number of statistically significant differences than could be established when using 2D methods. Moreover, by carefully delimiting the 3D structures under analysis using the 3D VR system, we were able to reveal a time dependent loss in colocalization between the Tau and microtubule network as an early event in neuronal injury. This behavior could not be reliably detected using a 2D based projection. We conclude that, using 3D colocalization analysis, biologically relevant samples can be interrogated and assessed with greater precision, thereby better exploiting the potential of fluorescence-based image analysis in biomedical research.

## Introduction

Fluorescence microscopy allows the tagging of particular macromolecules of interest and the subsequent study of their role in biological processes [1, 2]. With advances in fluorescence image acquisition and processing, such as confocal and Super Resolution Structured Illumination Microscopy (SR-SIM), it has become common practice to analyze the z-stacks, consisting of multiple two-dimensional (2D) fluorescence image frames, which these microscopes produce. These 2D images can be three-dimensionally (3D) reconstructed using direct-volume rendering [3–6], allowing the interrogation of the sample in 3D which can provide additional insight into biological events.

In the biological sciences, the interaction of two molecular structures or proteins is often of key interest, and hence colocalization analysis is commonly performed. Colocalization refers to the geometric codistribution of two fluorescent labels or color channels. It is usually assessed over a whole cell and metrically described using Pearson’s correlation coefficient (PCC), Manders’ overlap coefficient (MOC) and Manders’ correlation coefficient (MCC). In order to perform accurate colocalization analysis, it is important to precisely identify and select the region of interest (ROI) over which the colocalization will be calculated. The ROI is a sub-volume of the sample that is considered in the analysis. However, the precise selection of the ROI within a sample of interest, in 3D space, remains a practical challenge. Although the z-stack is inherently 3D, most established colocalization analysis methods define the ROI on a 2D projection of the z-stack, usually the maximum intensity projection (MIP). The MIP is a data visualization method in which the voxel with the maximum intensity along the view direction is projected onto a 2D plane. In the case of a z-stack, the view direction is usually aligned with the z-axis. Because the projection is usually orthographic, at each x-y coordinate the MIP retains only the voxel among all the image frames with the maximum intensity. Using this projection, however, excludes most of the data from the analysis. Furthermore, complex 3D structures cannot, in general, be adequately selected on the 2D projected image. Consequently, the use of the MIP for ROI selection can result in both the exclusion of relevant data points and the inclusion of irrelevant data, leading to possible inaccuracies in the colocalization analysis.

Often, the precise positioning analysis of subcellular structures is hampered by the limitations of current ROI selection methods. It has been shown that a 3D mask can be used to make a 3D selection [7–9]. The most common way this is done is by drawing boundary lines around the sample to create a mesh. This mesh is then allowed to shrink until it fits closely around the structure requiring analysis, after which every data point inside the mesh forms part of the ROI [10]. This approach works well for clearly defined regions, but it is not interactive and does not easily extend to complex selection combinations. Moreover, when using conventional 3D reconstruction and visualization of a sample, this reconstruction is ultimately displayed on a 2D screen which often leads to ambiguous data interpretation, especially when the 3D structure in the sample is complex. An inaccurate interpretation of the visualization can lead to inaccuracies in the selection, since it is not immediately apparent how well the 3D selection matches the desired ROI, without constantly rotating the sample, or providing several view angles.

We employ a recently developed virtual reality (VR) system that allows sample visualization and ROI selection in true 3D, thereby achieving an unambiguous representation of the data [11]. Since more data points are taken into account when using a 3D ROI, the statistical uncertainty associated with the colocalization metrics will also be reduced [12, 13]. We use this VR-assisted 3D ROI selection method to analyze a neuronal cell that is obtained using SR-SIM. By selecting regions and subcellular structures of biological relevance, such as the perinuclear, middle and membrane regions and performing extensive colocalization analysis, we demonstrate the improved precision and control achieved by the 3D ROI selection. Neuronal cells have been chosen as the subject of our study due to the particularly high relevance of accurate ROI control to their analysis. By assessing distinct structures such as the tubulin network and protein aggregates, we demonstrate how the 3D ROI selection can enhance our understanding of biological processes. Specifically, we have assessed the dissociation between the protein Tau and the microtubulin network in an *in vitro* model of autophagy dysfunction, which mimics a key pathology in Alzheimer’s disease [14], by analyzing the colocalization of Tau and acetylated *α*-tubulin.

### Neuronal cell investigation

One of the key structures of interest in neurobiology is the microtubule network, not only due to its critical role in neuronal integrity and transport processes, but also due to its role in the development of neuronal pathology. Alzheimer’s disease is a neurodegenerative disorder of the brain and a leading cause of dementia. Severe cognitive and short term memory deficits are commonly associated with this disease. The pathology is characterized by protein aggregates that manifest in brain tissue due to a dysfunction in protein degradation systems. One such affected system is the autophagy lysosomal pathway (ALP), which is a major catabolic process in eukaryotic cells that is responsible for the degradation of long-lived or damaged proteins and organelles. This pathway relies on the microtubulin (MT) network for the transportation of autophagosomes, which are double-membraned structures that enclose the proteins or organelles destined for degradation, towards lysosomes, which are compartments (or vesicular structures) within cells that contain enzymes with acidic properties. The lysosomes fuse with the autophagosome and degrade the constituents of the vesicle, now known as an autophagolysosome. Disruption of the ALP is a known perturbation that occurs within pathogenesis of Alzheimer’s disease [15–17]. The deterioration in the breakdown of autophagolysosomes leads to their accumulation on the MT, which disrupts the normal transport of other vesicles such as mitochondria [18, 19]. The disruption of the ALP further leads to the accumulation of proteins that rely on this degradative system to maintain their turnover. Among these aggregates are the neurofibrillary tangles (NFTs) which are composed of the microtubule-associated protein Tau, that controls the stability of the microtubulin network [20]. MT acts as part of the cytoskeleton and platform for dynein dependent autophagosome transport [21]. The fluorescent labels used in our analysis make the Tau visible as a green signal and the MT (acetylated *α*-tubulin) visible as a red signal in the SR-SIM z-stack.

The relationship between autophagy dysfunction and microtubule stability, however, remains unclear. Therefore, the interaction between Tau and MT as well as their colocalization is of interest and was chosen as the focus of this study.

## Materials and methods

### Cell culture and transfections

GT1-7 murine hypothalamic cells were cultured in DMEM supplemented 10% FBS and 1% PenStrep at 37°C and 5% CO_2_ atmosphere and treated with chloroquine diphosphate. In order to functionally disrupt autophagy, a key feature in neurodegeneration, GT1-7 cells were exposed to 100 μM of CQ for 6 and 24 hours respectively. 50 000 cells were trypsinsed, counted and washed in PBS. Next, cells were resuspended in 100 μL sterile Neon^®^ Resuspension buffer containing 5 μg of GFP-Tau (P301L) DNA. The suspension was aspirated into a gold plated Neon^®^ Tip using a Neon^®^ Pipette and cells were electroporated at 1350 V for 1 pulse lasting 30 ms.

### Immunofluorescence and super resolution structured illumination microscopy (SR-SIM)

Transfected cells were seeded into 6-well dishes containing 5 mm round coverslips and 2 mL fresh media treated with chloroquine after 24 hours, followed by fixation for 10 minutes in a 1:1 ratio of 4% Paraformaldehyde: DMEM at 37°C. Cells were blocked using 3% donkey serum in PBS, followed by probing the required primary antibody, i.e. acetylated *α*-tubulin (Santa Cruz, 23950) overnight at 4°C. Next, cells were incubated with a secondary antibody, Alexa Fluor 568 (ThermoFisher, A-10042) for 50 minutes. Coverslips were then washed 3x5 minutes with PBS and using Dako^®^ fluorescent mounting media. For SR-SIM analysis of GFP-Tau transfected cells, thin (0.1 μm) z-stacks of high-resolution (1024x1024 pixels) image frames were collected by utilizing an alpha Plan-Apochromat 60x/1.4 oil immersion DIC M27 ELYRA objective on an ELYRA PS.1 system (Carl Zeiss Microimaging, Germany) equipped with a 488nm laser (100mW) in 5 rotations, 561nm laser (100mW) in 5 rotations and an Andor EM-CCD camera (iXon DU 885). Images were reconstructed using Zeiss Zen Black Software (2012) based on a structured illumination algorithm [22]. Colocalization analysis of these reconstructed super-resolution images using both the 2D and 3D VR-assisted 3D ROI selection systems.

### Method

We performed viability assays to determine the degree of cell death after cells were exposed to chloroquine. Two points in time were chosen in order to mimic a mild and more severe neuronal injury, with significant cell death occurring only after 24 hours (S1 Fig). This would allow the assessment of the Tau-microtubulin interaction in a time and injury dependent manner and consequently the study of the molecular perturbations occurring prior to and at the onset of cell death (i.e. neurodegeneration). Using SR-SIM, cells were imaged at 0 hours (control, before chloroquine exposure) and again after 6 and 24 hours. We will henceforth refer to these points in time as *t* = 0, *t* = 6 and *t* = 24 respectively. Four representative cells were selected for analysis at each point in time. Hence our dataset consisted of a total of 12 cells. For each of these cells the z-stack consisted of 30 slice images on average.

**Fig 1:**
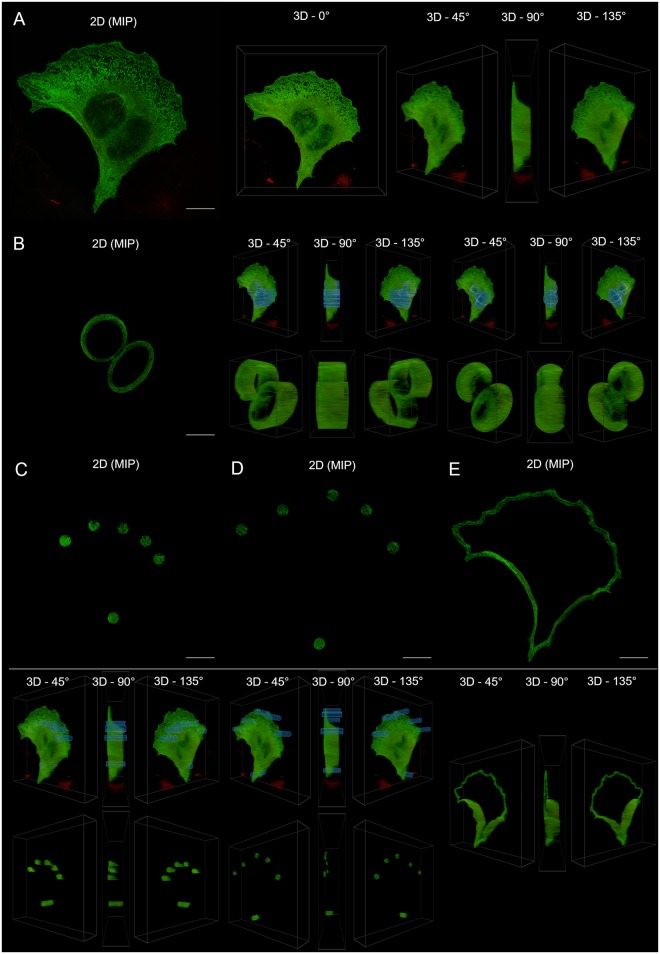
The main ROIs analyzed. Comparison of the MIP and the 3D visualization of some of the main regions analyzed for colocalization. The 3D image shows the selection overlaid on the whole cell, as well as the selection isolated from the remainder of the cell. The depicted cell is from the control sample set (*t* = 0). A: The whole cell. B: The perinuclear region for both a cylindrical and ellipsoidal selection. C: The sampled middle region. D: The sampled membrane region. E: The freehand membrane region (no overlay shown).

We hypothesized high levels of colocalization under healthy control conditions, with increasing dissociation between Tau and microtubulin and an associated loss of colocalization that reach their highest levels at *t* = 24. The colocalization was analyzed in both 2D (using the MIP) and 3D for the whole cell, the perinuclear region, the middle and membrane regions of the cell and a few prominent microtubulin strands and protein aggregates. These regions, shown in Fig 1, were chosen in order to determine the contribution of each to the colocalization profile of the cell as a whole.

#### Colocalization metrics

Colocalization was calculated by applying three metrics to the 3D z-stack. These metrics were Pearson’s Correlation Coefficient (PCC) [23, 24], Manders’ Overlap Coefficient (MOC) and Manders’ Correlation Coefficient (MCC) [25, 26]. The equations used to calculate these metrics are shown in Table 1.

**Table 1:** Colocalization metrics *R*_*i*_ represents the intensity of voxels in channel 1 (red fluorescent label) and *G*_*i*_ represents the intensity of voxels in channel 2 (green fluorescent label). $\bar{R}$ and $\bar{G}$ represent the mean intensities of the respective channels [26].

Colocalization Metric	Equation	Number
Pearson’s Correlation Coefficient	$\begin{array}{c} \text{PCC}=\frac{\sum_{i}\left(R_{i}-\bar{R}\right)\times \left(G_{i}-\bar{G}\right)}{\sqrt{\sum_{i}{\left(R_{i}-\bar{R}\right)}^{2}\times \sum_{i}{\left(G_{i}-\bar{G}\right)}^{2}}} \end{array}$	Eq 1
Manders’ Overlap Coefficient	$\begin{array}{c} \text{MOC}=\frac{\sum_{i}\left(R_{i}\times G_{i}\right)}{\sqrt{\sum_{i}R_{i}^{2}\times G_{i}^{2}}} \end{array}$	Eq 2
Manders’ Correlation Coefficient (Ch 1)	$\begin{array}{c} \text{M}1=\frac{\sum_{i}R_{i,colocal}}{\sum_{i}R_{i}}\\ \text{where}\;R_{i,colocal}=\{\begin{array}{cc} R_{i}, & \text{if}\;G_{i}>0\\ 0, & \text{if}\;G_{i}=0 \end{array} \end{array}$	Eq 3
Manders’ Correlation Coefficient (Ch 2)	$\begin{array}{c} \text{M}2=\frac{\sum_{i}G_{i,colocal}}{\sum_{i}G_{i}}\\ \text{where}\;G_{i,colocal}=\{\begin{array}{cc} G_{i}, & \text{if}\;R_{i}>0\\ 0, & \text{if}\;R_{i}=0 \end{array} \end{array}$	Eq 4

#### Colocalization visualization

It is standard practice to evaluate colocalization not only by means of metrics, but also by means of visualization. We therefore also visualized the colocalized volumes by either superimposing them as white voxels on the 3D sample, or by visualizing only the colocalized voxels in white or directly in their fluorescence emission spectrum colors [27, 28]. This served largely to assist in making ROI selections. To allow comparison, the colocalization was visualized on the MIP in a similar manner.

#### ROI selection tools

**Fig 2:**
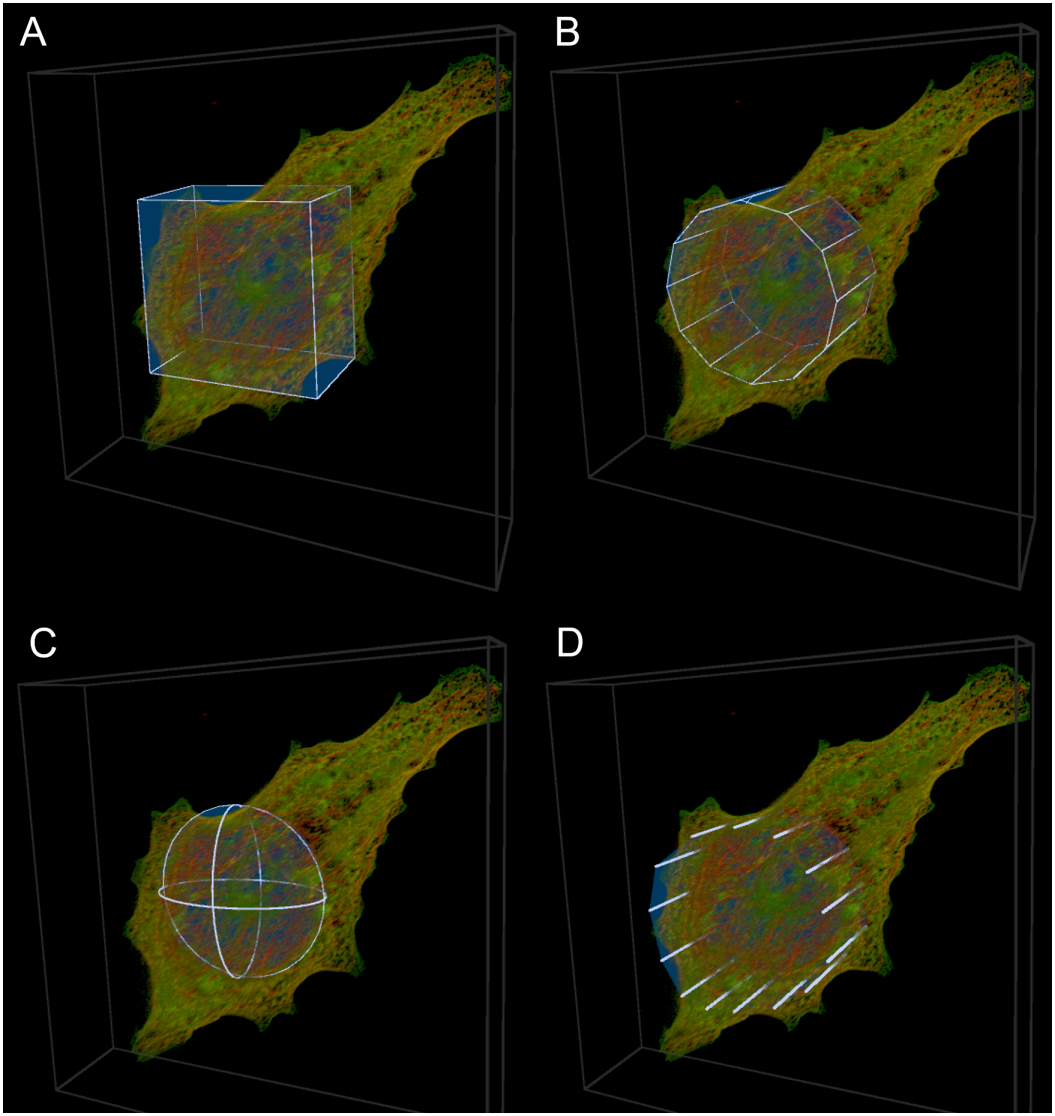
ROI selection tools. The ROI selection tools employed in the analysis. Combinations of these tools were used to make selections. A: The box tool. B: The cylinder tool. C: The sphere tool. D: The freehand tool.

Using the VR system, the following 3D selection tools were employed: *box*, *cylinder*, *sphere* and *freehand* (Fig 2), or any combination thereof. We will henceforth refer to each application of a tool as a selection. For the freehand selection, ROIs were traced out in 3D using either head movement and a gamepad or alternatively by pointing and drawing with the index finger using a hand tracking system, as has been demonstrated before [11]. It was possible to isolate and enlarge a selection, such as a single tubulin strand, and subsequently to refine this selection. In this way smaller, more precisely defined, sections of the sample could be visualized and analyzed independently. For a demonstration of this process in practice see S1 Video.

It was also possible to remove the inner part of a selection by using selections within selections, which provided greater versatility. Advantage was taken of this when selecting the perinuclear region of a cell, where the inside of the nucleus needed to be excluded from the ROI by placing a smaller ellipsoid within a larger ellipsoid and excluding the overlapping volume (Fig 1B).

In order to directly compare the 3D and MIP approaches, the closest comparable 2D ROI was used when calculating colocalization from the MIP. When selecting the perinuclear region, for example, the 3D ROI was an ellipsoid within an ellipsoid, which is most closely represented by an ellipse within an ellipse in 2D. Since an ellipse within an ellipse on the MIP corresponds to an elliptic cylinder within an elliptic cylinder in 3D, we see that certain selections are only accurately possible when specified directly in three dimensions.

#### Analysis

For the colocalization analysis, the following six ROIs were chosen: the whole cell, the perinuclear region, six to eight evenly spaced ROIs in both the middle and membrane regions of the cell, the entire membrane selected continuously using the freehand tool, microtubulin strands and protein aggregates. In order to gain insight into the effect of the 3D selection geometry on the colocalization results, both an elliptic cylinder and an ellipsoid were used when making 3D ROI selections. This is especially important for the perinuclear region where the underlying structure resembles an ellipsoid.

A total of four representative cells at each considered time (*t* = 0, *t* = 6 and *t* = 24) were analyzed for colocalization using both the 3D and the 2D MIP approaches to ROI selection. When comparing analysis results between the 3D and 2D selections, we will refer to a comparison of the *ROI selection methods*. For each point in time the mean of each colocalization metric over the four cells was calculated. A 95% confidence interval was calculated for each mean based on the t-distribution. Since four samples per group were analyzed for each estimate of the mean, the associated *z* value for the 95% confidence interval is 2.776. Note that the confidence interval was not used to determine significant differences but only as an indication of the spread of the data. Confidence intervals were calculated as follows (where *s* is the sample standard deviation):

$$\begin{array}{c} {\text{CI}}_{95\%}=\bar{x}\pm 2.776\times \frac{s}{\sqrt{4}} \end{array}$$ (5)

For the sampled (cylinder and ellipsoid) membrane regions at *t* = 24, the sample size was only two and not four, since in two cases the cell had already shrunk to a point at which the membrane could no longer be distinguished from the middle region. Accordingly, for this case the confidence interval’s *z* value was adjusted to 4.303.

Protein aggregates were most clearly visible after 6 hours, and were therefore only analyzed at *t* = 6.

In order to determine whether there was a statistically significant difference between calculated colocalization, either at two points in time or when using different ROI selection methods (i.e. 2D vs 3D), we first used the f-test to determine the equality of the two associated variances and then used this result to select the appropriate two-sample, two-tailed t-test. The p-value obtained from this t-test could then be used to determine statistically significant differences (*p* < 0.05). In all cases the null hypothesis (*H*_*o*_) was that the two calculated colocalization metrics were the same.

## Results

**Fig 3:**
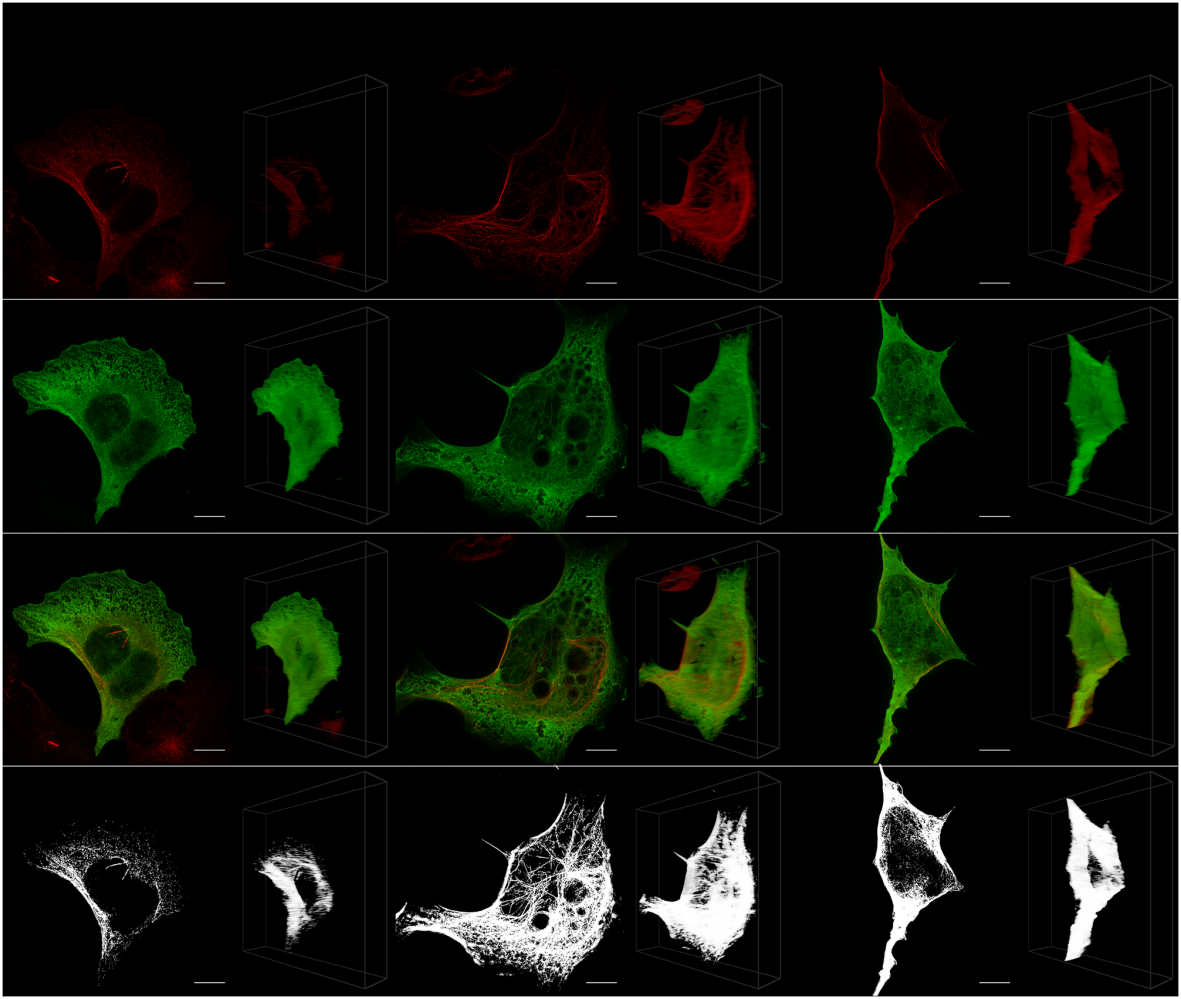
Whole cell. Representative cells for each considered time (*t* = 0, *t* = 6, *t* = 24) with the maximum intensity projection on the left and the 45° 3D view on the right. Shown from top to bottom are the acetylated *α*-tubulin (red), the Tau (green), the tubulin and Tau overlaid, and only the colocalized pixels/voxels.

**Fig 4:**
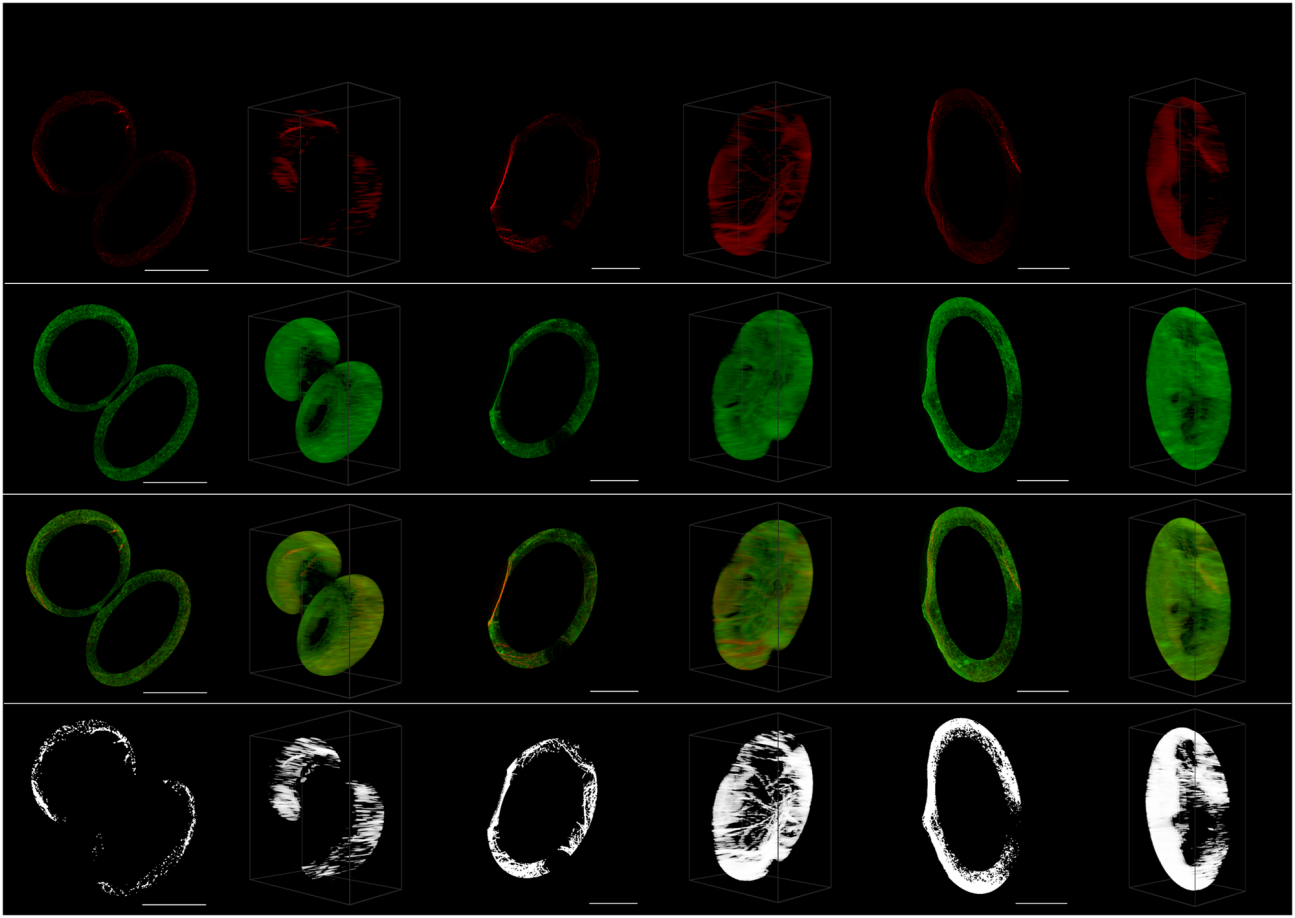
Ellipsoidal perinuclear region. Representative cells for each considered time (*t* = 0, *t* = 6, *t* = 24) with the maximum intensity projection on the left and the 45° 3D view on the right. Shown from top to bottom are the acetylated *α*-tubulin (red), the Tau (green), the tubulin and Tau overlaid, and only the colocalized pixels/voxels.

**Fig 5:**
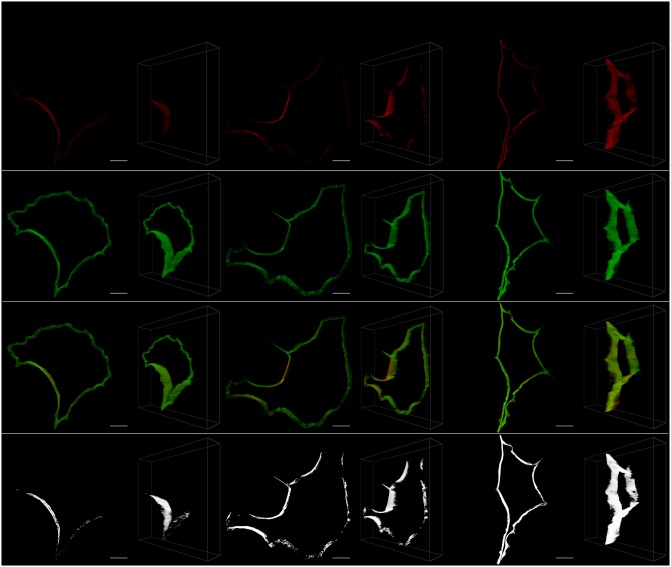
Freehand membrane region. Representative cells for each considered time (*t* = 0, *t* = 6, *t* = 24) with the maximum intensity projection on the left and the 45° 3D view on the right. Shown from top to bottom are the acetylated *α*-tubulin (red), the Tau (green), the tubulin and Tau overlaid, and only the colocalized pixels/voxels.

**Fig 6:**
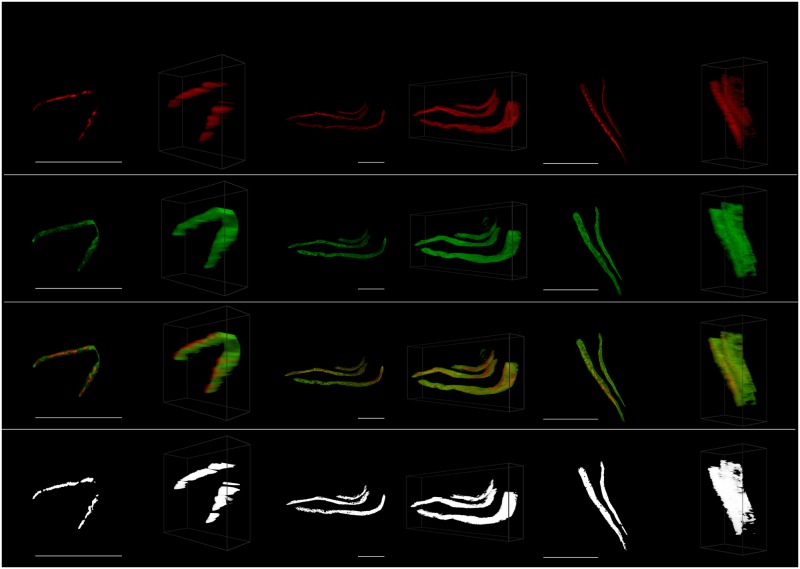
Microtubulin strands. Representative cells for each considered time (*t* = 0, *t* = 6, *t* = 24) with the maximum intensity projection on the left and the 45° 3D view on the right. Shown from top to bottom are the acetylated *α*-tubulin (red), the Tau (green), the tubulin and Tau overlaid, and only the colocalized pixels/voxels.

**Fig 7:**
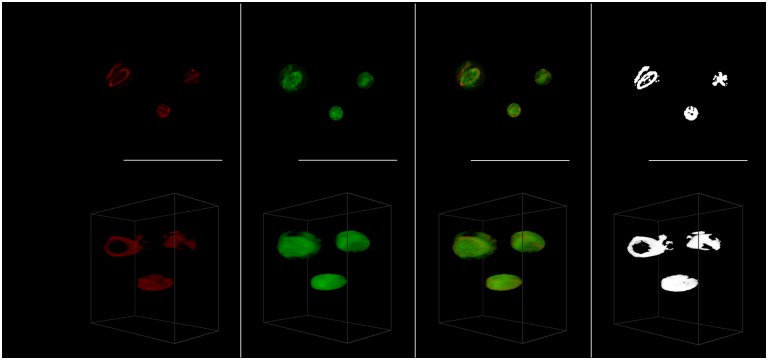
Protein aggregates. Representative cell for *t* = 6 with the maximum intensity projection at the top and the 45° 3D view with the ROI isolated from the whole cell at the bottom. Shown from left to right the acetylated *α*-tubulin (red), the Tau (green), the tubulin and Tau overlaid, and only the colocalized pixels/voxels. For the protein aggregates only the results at *t* = 6 were visualized and analyzed.

Figs 3–7 visualize representative cells for each considered point in time using both a 2D MIP and 45° 3D view. Note that a single 3D view cannot adequately convey a true perception of the cell structure as perceived in virtual reality. In order to conserve space, only the view that generally best highlights the strength of the 3D approach is shown. For a better understanding of what is perceived in VR, the reader is referred to S1 Video.

### PCC and MOC results

Tables 2 and 3 show that, for all 3D ROI selections, there is a decrease in the colocalization calculated using PCC and MOC at both *t* = 6 and *t* = 24 relative to the control, except for the microtubulin selection at *t* = 6 when using PCC. At *t* = 6, statistically significant differences relative to the control were observed for five of the nine selections (excluding the protein aggregates) for PCC (p-values < 0.032) and seven of the nine for MOC (p-values < 0.044). At *t* = 24, statistically significant differences relative to the control were observed for five of the nine selections for PCC (p-values < 0.038) and two of the nine for MOC (p-values < 0.049).

**Table 2:** Pearson’s correlation coefficients calculated for Tau and acetylated *α*-tubulin in different areas of GT1-7 cells under control conditions and after exposure to chloroquine for 6 and 24 hours respectively. (Data are presented as mean ± 95% confidence interval). * indicates significant differences relative to control (*p* < 0.05); A shaded block indicates that the ROI selection methods (i.e. 3D vs 2D) are statistically significantly different. In all cases statistical significance was determined using the two-sample, two-tailed t-test. Protein aggregates were only analyzed at the 6 hour time point.

	3D			2D (MIP)		
	Control (*t* = 0)	6 Hour (*t* = 6)	24 Hour (*t* = 24)	Control (*t* = 0)	6 Hour (*t* = 6)	24 Hour (*t* = 24)
**Whole Cell**	0.72 ± 0.07	**0.60** ± 0.07*	0.65 ± 0.10	0.67 ± 0.16	0.62 ± 0.12	0.74 ± 0.08
**Perinuclear** (Cylinder)	0.65 ± 0.07	**0.47** ± 0.09*	**0.47** ± 0.12*	0.58 ± 0.25	0.77 ± 0.07	0.79 ± 0.06
**Perinuclear** (Ellipsoid)	0.66 ± 0.07	**0.43** ± 0.10*	**0.45** ± 0.14*			
**Middle Region** (Cylinder)	0.65 ± 0.10	0.50 ± 0.13	**0.40** ± 0.13*	0.55 ± 0.31	0.79 ± 0.05	0.80 ± 0.10
**Middle Region** (Ellipsoid)	0.52 ± 0.10	**0.36** ± 0.07*	0.31 ± 0.19			
**Membrane** (Cylinder)	0.75 ± 0.07	**0.56** ± 0.16*	**0.48** ± 0.24*	0.68 ± 0.16	0.78 ± 0.08	0.85 ± 0.07
**Membrane** (Ellipsoid)	0.61 ± 0.13	0.42 ± 0.14	0.42 ± 0.33			
**Membrane** (Freehand)	0.74 ± 0.06	0.59 ± 0.16	**0.58** ± 0.09*	0.69 ± 0.12	0.73 ± 0.10	0.80 ± 0.05
**Microtubulin**	0.46 ± 0.15	0.47 ± 0.05	0.41 ± 0.07	0.29 ± 0.29	**0.78** ± 0.05*	**0.82** ± 0.05*
**Protein Aggregates**		0.25 ± 0.16			0.82 ± 0.04	

**Table 3:** Manders’ overlap coefficient with procedure described in Table 2.

	3D			2D (MIP)		
	Control (*t* = 0)	6 Hour (*t* = 6)	24 Hour (*t* = 24)	Control (*t* = 0)	6 Hour (*t* = 6)	24 Hour (*t* = 24)
**Whole Cell**	0.76 ± 0.05	**0.66** ± 0.07*	0.67 ± 0.10	0.79 ± 0.08	0.76 ± 0.06	0.77 ± 0.07
**Perinuclear** (Cylinder)	0.80 ± 0.04	**0.65** ± 0.10*	**0.67** ± 0.10*	0.86 ± 0.05	0.78 ± 0.08	0.80 ± 0.06
**Perinuclear** (Ellipsoid)	0.81 ± 0.04	**0.65** ± 0.07*	**0.67** ± 0.09*			
**Middle Region** (Cylinder)	0.81 ± 0.05	**0.69** ± 0.08*	0.66 ± 0.13	0.89 ± 0.03	**0.80** ± 0.05*	0.80 ± 0.10
**Middle Region** (Ellipsoid)	0.81 ± 0.06	**0.69** ± 0.07*	0.66 ± 0.13			
**Membrane** (Cylinder)	0.82 ± 0.04	**0.69** ± 0.11*	0.71 ± 0.22	0.88 ± 0.03	**0.79** ± 0.08*	0.86 ± 0.07
**Membrane** (Ellipsoid)	0.82 ± 0.05	**0.69** ± 0.11*	0.73 ± 0.21			
**Membrane** (Freehand)	0.80 ± 0.02	0.68 ± 0.14	0.72 ± 0.09	0.84 ± 0.05	0.75 ± 0.09	0.81 ± 0.05
**Microtubulin**	0.71 ± 0.13	0.67 ± 0.06	0.67 ± 0.09	0.77 ± 0.14	0.79 ± 0.05	0.83 ± 0.05
**Protein Aggregates**		0.69 ± 0.03			0.82 ± 0.04	

Table 2 also shows that, when the colocalization is calculated using a MIP ROI selection, there is an increase in PCC at both *t* = 6 and *t* = 24 relative to the control, except when selecting the whole cell at *t* = 6. On the other hand, Table 3 shows a decrease in MOC relative to the control for the same MIP selections for all regions from the control to *t* = 6 and control to *t* = 24, except for the microtubulin which showed an increase. None of these differences were statistically significant, however, except for the microtubulin PCC values at both *t* = 6 and *t* = 24 and the MOC for the middle and membrane regions at *t* = 6. No consistent change in PCC or MOC could be determined from *t* = 6 to *t* = 24, and none of these differences were statistically significant.

When considering the differences in PCC calculated using the 2D MIP and the 3D ROI selections, we find that there is no statistically significant difference between any of the selections at *t* = 0. For both *t* = 6 and *t* = 24, however, PCC indicated lower colocalization for all 3D selections. At *t* = 6 and *t* = 24, respectively, eight of the ten (p-values < 0.022) and and six of the nine (p-values < 0.003) differences were statistically significant. The MOC values were also lower when using the 3D ROIs than when using the MIP ROIs at all three considered points in time. At *t* = 0, the MOC of the 3D ROIs were statistically significantly lower than the MIP ROIs for three of the nine selections (p-values < 0.028). At *t* = 6 and *t* = 24, the MOC of the 3D ROIs were statistically significant lower than the MIP ROIs for six of the ten (p-values < 0.046) and three of the nine (p-values < 0.05) selections, respectively.

### MCC results

The results of the colocalization analysis based on Manders’ correlation coefficient (MCC) are compiled in Tables 4 and 5. We will refer to the MCC calculation for Tau simply as M1 and to the MCC calculation for acetylated *α*-tubulin simply as M2. When using 3D ROI selections, M1 showed an average increase in colocalization with time for the whole cell, for the membrane and for the microtubulin. However, at *t* = 24 only the whole cell (*p* = 0.023) and the freehand membrane selection (*p* = 0.026) had increased significantly relative to the control. In contrast, M2 showed an average decrease in colocalization from *t* = 0 through *t* = 6 to *t* = 24 for all 3D ROI selections except the cylindrical membrane selection at *t* = 6 and both membrane selections at *t* = 24. However, none of these decreases were statistically significant. When using MIP ROI selections, there was practically no variation in either M1 or M2, with most selections leading to a calculated MCC value of one. There were therefore no statistically significant differences between the values of M1 and M2 calculated between any of the three time instances *t* = 0, *t* = 6 and *t* = 24 when using the MIP-derived ROIs.

**Table 4:** Manders’ correlation coefficient for Tau (M1) with procedure described in Table 2.

	3D			2D (MIP)		
	Control (*t* = 0)	6 Hour (*t* = 6)	24 Hour (*t* = 24)	Control (*t* = 0)	6 Hour (*t* = 6)	24 Hour (*t* = 24)
**Whole Cell**	0.84 ± 0.09	0.91 ± 0.06	**0.95** ± 0.03*	0.996 ± 0.004	0.998 ± 0.002	0.999 ± 0.001
**Perinuclear** (Cylinder)	0.96 ± 0.02	0.94 ± 0.02	0.96 ± 0.03	1.00 ± 0.00	1.00 ± 0.00	1.00 ± 0.00
**Perinuclear** (Ellipsoid)	0.97 ± 0.01	0.94 ± 0.03	0.96 ± 0.04			
**Middle Region** (Cylinder)	0.94 ± 0.04	0.93 ± 0.04	0.96 ± 0.02	1.00 ± 0.00	1.00 ± 0.00	1.00 ± 0.00
**Middle Region** (Ellipsoid)	0.97 ± 0.03	0.96 ± 0.03	0.97 ± 0.02			
**Membrane** (Cylinder)	0.85 ± 0.06	0.91 ± 0.04	0.96 ± 0.01	1.00 ± 0.00	1.00 ± 0.00	1.00 ± 0.00
**Membrane** (Ellipsoid)	0.91 ± 0.04	0.95 ± 0.04	0.97 ± 0.02			
**Membrane** (Freehand)	0.84 ± 0.09	0.91 ± 0.05	**0.96** ± 0.03*	0.999 ± 0.001	1.00 ± 0.00	1.00 ± 0.00
**Microtubulin**	0.92 ± 0.10	0.93 ± 0.04	0.97 ± 0.03	1.00 ± 0.00	1.00 ± 0.00	1.00 ± 0.00
**Protein Aggregates**		0.98 ± 0.02			1.00 ± 0.00	

**Table 5:** Manders’ correlation coefficient for acetylated *α*-tubulin (M2) with procedure described in Table 2.

	3D			2D (MIP)		
	Control (*t* = 0)	6 Hour (*t* = 6)	24 Hour (*t* = 24)	Control (*t* = 0)	6 Hour (*t* = 6)	24 Hour (*t* = 24)
**Whole Cell**	0.85 ± 0.04	0.82 ± 0.08	0.76 ± 0.13	0.92 ± 0.06	0.97 ± 0.02	0.77 ± 0.15
**Perinuclear** (Cylinder)	0.94 ± 0.04	0.88 ± 0.04	0.90 ± 0.04	0.999 ± 0.001	0.996 ± 0.004	0.998 ± 0.002
**Perinuclear** (Ellipsoid)	0.92 ± 0.06	0.85 ± 0.07	0.88 ± 0.04			
**Middle Region** (Cylinder)	0.94 ± 0.03	0.89 ± 0.07	0.88 ± 0.07	0.999 ± 0.001	0.998 ± 0.02	0.999 ± 0.001
**Middle Region** (Ellipsoid)	0.96 ± 0.04	0.91 ± 0.06	0.89 ± 0.07			
**Membrane** (Cylinder)	0.85 ± 0.06	0.87 ± 0.05	0.92 ± 0.10	0.99 ± 0.01	0.998 ± 0.002	1.00 ± 0.00
**Membrane** (Ellipsoid)	0.92 ± 0.06	0.90 ± 0.07	0.93 ± 0.09			
**Membrane** (Freehand)	0.86 ± 0.05	0.83 ± 0.05	0.90 ± 0.06	0.98 ± 0.01	0.99 ± 0.01	0.98 ± 0.02
**Microtubulin**	0.95 ± 0.08	0.91 ± 0.04	0.94 ± 0.03	1.00 ± 0.00	0.999 ± 0.001	0.999 ± 0.001
**Protein Aggregates**		0.92 ± 0.06			0.99 ± 0.01	

Of the M1 values shown in Table 4, 21 of the 28 MCC values were significantly higher (p-values < 0.046) when selecting ROIs in 2D than when selecting ROIs in 3D. For the M2 values shown in Table 5, 19 of the 28 MCC values were significantly higher (p-values < 0.047) in 2D compared to the 3D selections.

## Discussion

When PCC and MOC were calculated using 3D ROI selections, all the values decreased after 6 and 24 hours relative to the control (except for the microtubulin at *t* = 6), with most of these decreases being statistically significant. These results support the notion that increased neuronal toxicity, i.e. autophagy dysfunction and protein aggregation, is associated with a loss of colocalization between Tau and tubulin, leading to a loss in microtubule stability. However, these decreases were not detected for PCC when using the 2D ROI selections. Instead, in this case, all selections showed average increases in colocalization, with the microtubulin (Fig 6) showing a statistically significant increase (p-values < 0.026) at both *t* = 6 and *t* = 24 relative to the control. This is contrary to both the expected behavior and the indication of the 3D analysis that there is no significant change in PCC for the microtubulin. The result of the 2D ROI selection is regarded as a false positive. Generally, 3D ROI selections must lead to a statistically more reliable estimate of colocalization, since the entire sample is used in the calculation instead of just a projection. Since the MIP is based on maximum values along the projected dimension, it also typically leads to higher colocalization estimates than when using the 3D ROI. This indicates a risk of false positives for 2D colocalization analysis. In contrast to PCC, MOC mostly indicated an average decrease in colocalization for both the 2D and 3D selections, where decreases observed for the middle and the cylindrical membrane regions were statistically significant. This seems to indicate that MOC is less affected by the limitations of a 2D projection than PCC.

For PCC, none of the differences between 2D and 3D ROI selections at the control (*t* = 0) were significant. This can be attributed to the initial (in the control condition) fairly good correlation between the colocalization of Tau and MT, which leads to a projection that is a good approximation of the true 3D colocalization. However, as soon as the cell is exposed to chloroquine and the neuronal toxicity increases, Tau and MT may start to dissociate in such a way that, when the volume is projected, PCC indicates a greater colocalization than when considered in three-dimensional space. However, at *t* = 0 MOC and MCC were observed to be more sensitive than PCC and it was possible to determine some statistically significant differences between the 3D ROI selections when compared with the 2D MIP selections (Tables 3–5). Since the 3D analysis consistently showed these differences, it can be regarded as a more robust analysis approach than the 2D MIP analysis.

The strength of the 3D VR-enabled system is most clearly demonstrated in the case of the 3D perinuclear selection (Fig 4), which is one of the more complex structures in the cell that was considered. In this case we compared how ellipsoidal and cylindrical ROI selections influenced the differences between the colocalization calculated at the control and at later time points. As was expected, the ellipsoidal selection, which is not possible in 2D, provided stronger evidence against the null hypothesis (*H*_*o*_), which is that the two colocalization metrics are the same, with smaller p-values (PCC *t* = 6: 0.005; PCC *t* = 24: 0.018; MOC *t* = 6: 0.004; MOC *t* = 24: 0.015) compared to the cylindrical selection (PCC *t* = 6: 0.009; PCC *t* = 24: 0.020; MOC *t* = 6: 0.014; MOC *t* = 24: 0.032). We believe that this is because more relevant data is taken into account when using the ellipsoidal ROI selection, especially at the top and bottom of the perinuclear region. A comparison of these selections is shown in Fig 1. In general, making selections in 3D increases the ability to detect differences in both PCC and MOC and we note specifically that no significant decrease was observed in PCC when using the traditionally considered MIP.

The analysis of MCC reveals that, when calculated using 2D MIP, many colocalization estimates equal unity. When the MIP is generated, only the maximum intensities are projected and therefore the less-colocalized (lower) intensities are lost. This leads to the summation of the same intensities in the numerator and in the denominator of Eqs 3 and 4 and a resultant MCC of unity. When making the same selections in 3D, all voxels, including those which are dimmer and considered less-colocalized, are taken into account, leading to variations in the calculated MCC. This indicates that the 3D metric is more sensitive to variation in the data and that, by performing the analysis in 3D rather than in 2D, colocalization in different regions can be compared.

For M1, an average increase was observed for most 2D and 3D ROI selections, with those for the whole cell (Fig 3) and the freehand membrane (Fig 5) selections at *t* = 24 being statistically significant (p-values < 0.026). This increase is in conflict with the decrease that was hypothesized and also calculated using the other metrics. We attribute this to an increase in the fluorescence brightness observed in the Tau fluorochrome at *t* = 24, which leads to a greater number of pixels having an intensity exceeding the predetermined colocalization thresholds. This increase in fluorescence intensity of the fluorochromes after long-term exposure (i.e. 24 hours) to chloroquine is likely not due to an increase in mean Tau intensity, but rather due to toxicity-induced cell shrinkage. This shrinkage leads to an increased fluorochrome concentration within cell and thus an increase in brightness. This problem was specific to our setup and should not be taken as a general weakness of MCC.

It is worth pointing out that no significant difference between the colocalization at *t* = 6 and *t* = 24 were found for any ROI selection for any of the considered metrics. From a biomedical perspective this is important, since only prolonged exposure to chloroquine led to significant cell death induction. This suggests that loss of colocalization between Tau and MT may be an early event in neuronal injury, prior to the onset of cell death, which has not previously been shown. The dysfunction of Tau has however previously been considered to contribute to the collapse of the cytoskeleton [29]. This result, therefore, contributes to the understanding of the molecular pathology that unfolds in the pathogenesis, which sheds light on disease mechanisms, but may also be useful for unraveling novel therapeutic targets, although more research is needed.

When comparing all the results obtained for the 2D and 3D ROI selections we note that most are statistically significantly different (especially in the case of MCC). From this we conclude that the result of a colocalization analysis is not only dependent on the metrics that are used, but also on the method of ROI definition. The results show that generally the 3D system is more sensitive than the 2D system and can decrease the occurrence of false positives and increase the sensitivity of the metrics.

Since 3D ROI selections include more data (voxels) than ROIs based on 2D MIPs, the resultant colocalization metrics should deliver more robust results. Therefore, calculating a colocalization estimate using an ROI defined in 3D offers greater confidence in the accuracy of the estimate than when it is defined in 2D on a MIP. For the same reason greater confidence is attributed to the estimates obtained using the freehand membrane selection over the cylindrical or ellipsoidal samples taken on the membrane, since the entire membrane is included in the calculation, instead of only samples along the membrane.

Using the 3D VR ROI selection system, specific regions could be analyzed. These were the perinuclear region (Fig 4), the cell’s middle regions (Fig 1), the cell membrane (Figs 1 and 5), the tubulin network (Fig 6) as well as protein aggregates (Fig 7). This 3D dissection of many cells allows greater precision of analysis. Since all selections and calculations remain in 3D, it leads to an increased resolving power of the colocalization analysis. This stands in contrast to typically only analyzing the whole-cell colocalization using MIPs (Fig 3).

Using our 3D VR-assisted system, we were able to isolate very fine sub-cellular structures such as protein aggregates and microtubulin strands through precise ROI selection. This allowed colocalization analysis to be considered specifically within those structures. This is not possible using conventional 2D analysis. These structures are visualized in Figs 6 and 7.

Although it has been previously shown that chloroquine treatment enhances Tau aggregation [30] while wild type and phosphomutant Tau species do not aggregate in autophagy-deficient cells [31], the colocalization profile under autophagy dysfunction has not previously been described in such precise and region specific manner. Here we describe a highly region-specific decrease in colocalization upon autophagy disruption, which, to our knowledge, has not been described previously. Similar results, albeit without region specific quantitative colocalization analysis, have been reported in models of Tauopathy, with a distinct redistribution pattern of Tau and the microtubule network in relation to the point of dendritic degeneration [32]. This stresses the importance of being able to perform highly defined, region-specific colocalization analysis, enabling the dissection of specific intracellular domains associated with neuronal pathology.

## Conclusion

Despite the advances in fluorescence microscopy that allow detailed 3D datasets of cells to be generated, most colocalization analysis is still performed using 2D MIPs. Our experiments indicate that enhanced and more sensitive colocalization analysis is possible when carrying out three-dimensional region of interest selection. We focused on neuronal cells, specifically investigating the dissociation between Tau and the microtubulin network in an *in vitro* model of autophagy dysfunction, mimicking a key pathology in Alzheimer’s disease, by analyzing the change in colocalization between Tau and acetylated *α*-tubulin at a control, 6 hour and 24 hour time points. After calculating several colocalization metrics using both the 2D and 3D ROI selection methods, we compared the colocalization estimated at the the different points in time on the basis of the 2D and 3D ROIs. Statistically significant decreases in colocalization over time were observed when using the 3D analysis, indicating that the dissociation of Tau and microtubulin may be an early event in neuronal injury prior to the onset of cell death. This tendency could not be consistently observed when using the 2D analysis. In fact, the 2D PCC analysis in some cases indicated increasing colocalization with time. Generally, differences observed in the 2D analysis were statistically less significant when compared with the 3D analysis.

We were able to show how very fine structures, such as protein aggregates and microtubulin strands, can be isolated and analyzed independently and with high precision. This paper is part of ongoing research in which the role of autophagy dysfunction in protein aggregation and tubulin stability in the context of neuronal toxicity is investigated, with the aim of discovering the key molecular mechanisms that govern neuronal fate. It is hoped that 3D virtual reality assisted ROI selection will allow the future analysis of samples of biological relevance can be interrogated with greater precision and control, thereby fully exploiting the potential of fluorescence-based image analysis in biomedical research.

## Supporting information

S1 Video Improved ROI selectivity demonstration video. In this video the virtual reality system that was used in this paper is demonstrated by selecting several regions of interest.
(MP4)

S1 Fig WST-1 reductive capacity assay of cells treated with CQ for 6 hours (A) and 24 hours (B), respectively. 100 *μ*M CQ was sufficient to cause a significant reduction in cell viability after 24 hours of CQ exposure, but not after 6 hours. All data are presented as a percentage of control (Mean ± SEM). ** (*p* < 0.05 vs control). N = 3 independent experiments.
(TIF)
