# Supplementary figures and images for: Improved region of interest selection and colocalization analysis in three-dimensional fluorescence microscopy samples using virtual reality
Source: PLoS One. 2018 Aug 29;13(8):e0201965. doi: 10.1371/journal.pone.0201965 (PMC6114514; doi:10.1371/journal.pone.0201965)

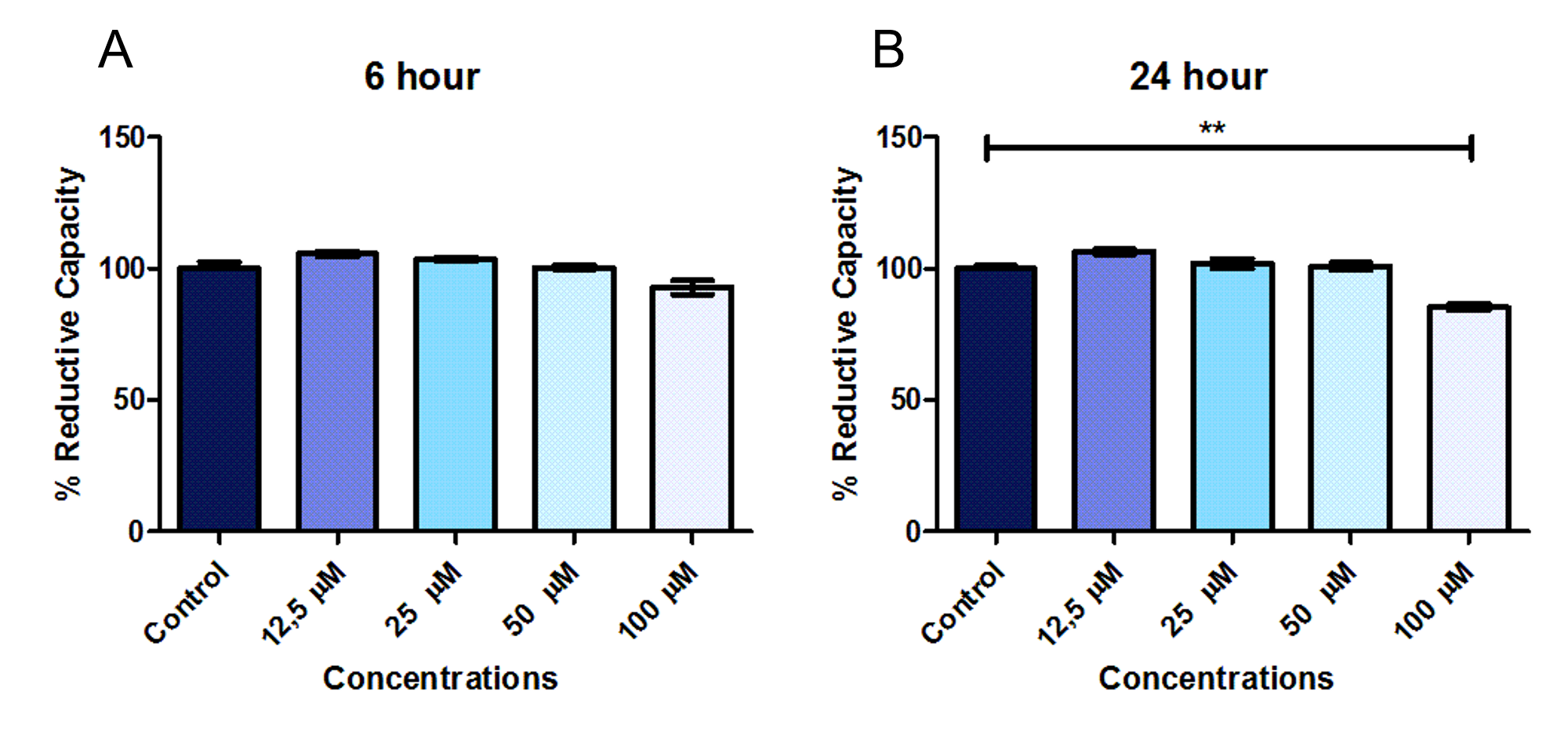

Supplement: S1 Fig — 100 μM CQ was sufficient to cause a significant reduction in cell viability after 24 hours of CQ exposure, but not after 6 hours. All data are presented as a percentage of control (Mean ± SEM). ** (p < 0.05 vs control). N = 3 independent experiments. (TIF) [file pone.0201965.s002.tif]
